# Tailor-Made Detection of Individual Phosphorylated and Non-Phosphorylated EPIYA-Motifs of *Helicobacter pylori* Oncoprotein CagA

**DOI:** 10.3390/cancers11081163

**Published:** 2019-08-13

**Authors:** Suneesh Kumar Pachathundikandi, Andrés Julián Gutiérrez-Escobar, Nicole Tegtmeyer

**Affiliations:** Department of Biology, Division of Microbiology, Friedrich Alexander University Erlangen-Nuremberg, Staudtstraße 5, D-91058 Erlangen, Germany

**Keywords:** CagA, *cag*PAI, dotblot, EPIYA motifs, *Helicobacter pylori*, signaling, type IV secretion, Abl and Src tyrosine kinases

## Abstract

The gastric pathogen and carcinogen *Helicobacter pylori* *(H. pylori)* encodes a type IV secretion system for translocation of the effector protein CagA into host cells. Injected CagA becomes tyrosine-phosphorylated at the five amino acid residue Glutamate-Proline- Isoleucine-Tyrosine-Alanine (EPIYA)-sequence motifs. These phosphorylated EPIYA-sites represent recognition motifs for binding of multiple host factors, which then manipulate signaling pathways to trigger gastric disease. Thus, efficient detection of single phosphorylated EPIYA-motifs in CagA is required. Detection of phospho-CagA is primarily performed using commercial pan-phosphotyrosine antibodies. However, those antibodies were originally generated to recognize many phosphotyrosines in various mammalian proteins and are not optimized for use in bacteria. To address this important limitation, we synthesized 11-mer phospho- and non-phospho-peptides from EPIYA-motifs A, B, and C, and produced three phospho-specific and three non-phospho-specific rabbit polyclonal CagA antibodies. These antibodies specifically recognized the corresponding phosphorylated and non-phosphorylated EPIYA-motifs, while the EPIYA-C antibodies also recognized the related East-Asian EPIYA-D motif. Otherwise, no cross-reactivity of the antibodies among EPIYAs was observed. Western blotting demonstrated that each EPIYA-motif can be predominantly phosphorylated during *H. pylori* infection. This represents the first complete set of phospho-specific antibodies for an effector protein in bacteria, providing useful tools to gather information for the categorization of CagA phosphorylation, cancer signaling, and gastric disease progression.

## 1. Introduction

The Gram-negative pathogen *Helicobacter pylori* (*H. pylori*) represents one of the most prevalent persistent bacterial infections in humans, affecting about half of the world population. The pathogen is associated with various gastric diseases, such as peptic ulceration, and represents the main risk factor for the development of gastric cancer [1,2,3,4]. A major disease-associated virulence factor is its type IV secretion system (T4SS) encoded by the cytotoxin-associated genes (*cag*) pathogenicity island (*ca*gPAI). The *ca*gPAI is present in the chromosome of highly virulent *H. pylori* strains but absent in less virulent isolates. This T4SS represents a syringe-like pilus device positioned in the bacterial membrane, and pilus formation is induced by contact with the host target cell [5,6]. The only known effector protein delivered by the *cag*T4SS to date is CagA [7,8,9]. CagA is about 125–145 kDa in size and shares no homology with any other protein in sequence databases. Remarkably, CagA seems to mimic a host protein [10] that hijacks the tyrosine phosphorylation machinery of the host cell [11]. Upon injection by the T4SS, CagA was shown to undergo tyrosine phosphorylation by Src [12,13] and Abl tyrosine kinases [14,15,16]. Subsequently, phosphorylated CagA (CagA^PY^) can bind a selection of cellular interaction partners with Src homology 2 (SH2) domains to manipulate host cell signaling. Currently, about 25 cellular binding partners have been identified that can interact with CagA in a phosphorylation-dependent or phosphorylation-independent manner, manipulating signal transduction pathways involved in cytoskeletal rearrangements, cell proliferation, cell motility, cell death, and inflammation to trigger gastric disease, including gastric cancer [17]. Due to these functions, CagA has been called the first bacterial oncoprotein that acts in mammalian cells [2].

The phosphorylation sites in CagA were identified by site-directed mutagenesis as the so-called EPIYA (Glu-Pro-Ile-Tyr-Ala)-motifs [13,18,19,20]. In *H. pylori* isolates from North America, Europe, Australia, and some Asian states (e.g., India and Malaysia), three different EPIYA-motifs have been classified as EPIYA-A, -B, and -C, depending on the neighboring sequence [2,7,21,22]. In isolates originating from East Asia (e.g., China, Korea and Japan), the EPIYA-C can be replaced by a related EPIYA-D site. Notable variability in the number and configuration of the EPIYA-sites has been observed in CagA proteins worldwide [2,16,21,22,23,24,25,26,27,28,29,30,31,32,33]. The importance of CagA phosphorylation for the bacteria is unknown; however, production of CagA^PY^ is required for the characteristic, so-called “scattering” or “elongation” phenotype, observed for *H. pylori*-infected cultured gastric epithelial cells, such as AGS (gastric adenocarcinoma) cells [13,18,34,35]. It was proposed that this phenotype has a key effect on *H. pylori* pathogenesis because it may impact various processes, such as wound healing, invasive growth, or metastasis of cancer cells, as well as immune responses in vivo [36,37,38].

Observations that CagA undergoes phosphorylation at tyrosine residues upon delivery into host cells have been mainly monitored by Western blotting and with commercial pan-phosphotyrosine antibodies [34,39,40,41,42]. These antibodies were developed to recognize multiple phosphotyrosine residues in mammalian cells, but were not specifically raised against bacterial proteins. Our recent studies identified six of those pan-phosphotyrosine antibodies, most notably PY-20, PY-99, and PY-100, recognizing both host cell proteins and phospho-CagA [43,44]. Using synthetic phospho- and non-phospho-peptides derived from each CagA EPIYA-motif, we have shown that a total of 9–11 amino acids containing the phospho-tyrosine residue in the EPIYA-motifs are necessary and sufficient for specific detection by these commercial antibodies, but the work revealed vast variability in sequence recognition [43,44]. In Western-type CagAs, three of the above antibodies recognized peptides of phosphorylated EPIYA-motifs A, B, and C equally well in vitro, whereas preferential binding to phosphorylated motif A, and motifs A and C, was found with two and one other antibodies, respectively [43,44]. However, systematic studies as to whether specific CagA EPIYA-sites can be detected by these antibodies during infection with *H. pylori* are not yet available, and based on the mentioned cross-reactivity might produce ambiguous results. Thus, the generation of phospho-specific antibodies for single EPIYA-motifs is urgently required. In the past, the generation of three respective rabbit antibodies was reported, which were produced against the peptide-based antigens RSVSPEPIpYATIDDL (EPIYA-motif C) [45], VGLSASPEPIpYAT (EPIYA-motif C) [5], and PEEPIpYTQVAK (EPIYA-motif B) [46]. However, these antibodies were not characterized in detail and a full set of phospho-specific EPIYA antibodies is not yet available. Here, we report the successful production of phospho-specific and non-phospho-specific polyclonal CagA antibodies raised against Western-type EPIYA-motifs A, B, and C, respectively, using 11-mer peptides as antigens. We confirmed the specificity of each antibody by Western blotting and demonstrate that the set of EPIYA-C antibodies also recognized the closely related EPIYA-D site. Subsequently, we performed infection experiments with Western- and East Asian-type *H. pylori* to study the patterns of CagA^PY^ following delivery into host cells. These data demonstrate for the first time that each of the CagA EPIYA-motifs can be phosphorylated upon infection. Our results are, therefore, highly valuable for detailed analysis of CagA phosphorylation events during infection and for determining their importance in signaling and gastric disease development by *H. pylori*.

## 2. Results

### 2.1. Phylogenetic Analysis of H. pylori CagA Carrying EPIYA-Motifs A, B, C and D

**Figure 1:**
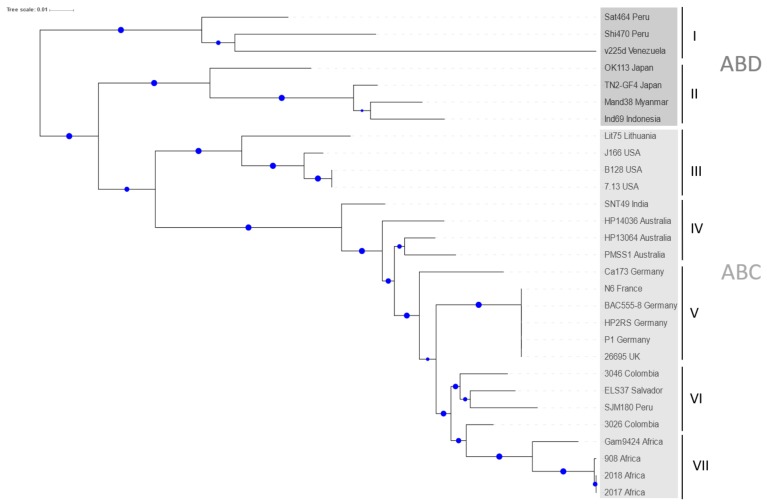
Neighbor-Joining tree of 29 non-redundant CagA protein sequences of patients from all continents. The strain names are indicated with the corresponding country when this information was available. Seven major clusters are identified and labelled with I to VII. Blue dots represent bootstrap values, the larger dots correspond to 100% and the smaller to 50%, respectively. The grayscale depicts ABD and ABC sequence types. The CagA protein sequences were downloaded from GenBank (Supplementary Table S1). For more information see text.

**Figure 2:**
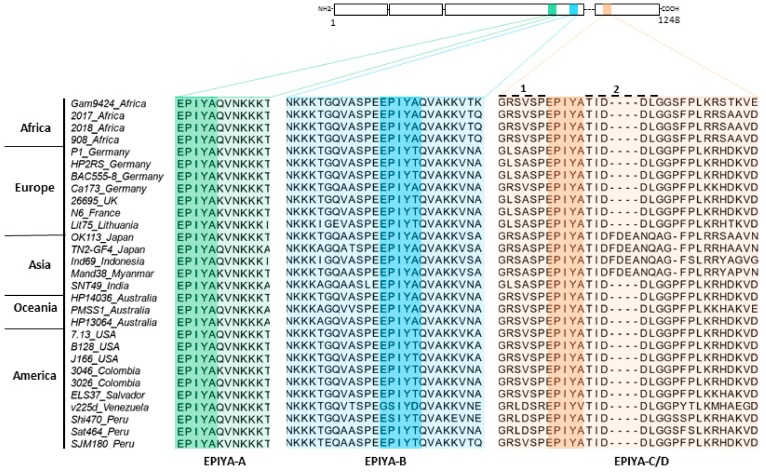
Sequence alignment of the EPIYA-motifs in CagA proteins from worldwide clinical *H. pylori* strains. Schematic representation of the CagA protein (top) and multiple alignment of a relevant segment (bottom) with color-shaded EPIYA-repeats A, B, and C/D. Variations in their flanking regions were found among clinical *H. pylori* isolates from different continents, as indicated above the alignment. The CagA protein sequences were downloaded from GenBank (Supplementary Table S1) and sequence alignment was done using ClustalW2 (http://www.ebi.ac.uk/Tools/msa/clustalw2/). For more information see text.

Currently, various major *H. pylori* populations have been described in tight relation with humans in Africa, Asia, Europe, and Sahul [47]. Many of the CagA proteins from these isolates contain the three EPIYA-sites A, B, and C/D. As a first step, the phylogeny of CagA-ABC/D types from 5 continents was investigated. The obtained tree separated protein sequences into two clusters: ABD and ABC (Figure 1). The ABD cluster is divided into Clades I and II, which are composed exclusively of hpEAsia strains. The ABC cluster is further divided into Clade III, which is composed of hpEurope strains, Clade IV, which contains strains from hpAsia2, Clade V, which is formed by hpEurope strains, Clade VI, which is composed of Latin-American strains closely related to the hpEurope population, and Clade VII, which contains the hpAfrica1 strains. The alignment shown in Figure 2 illustrates that CagA is characterized by a highly conserved set of amino acids located before and after each of the individual EPIYA-motifs A, B, and C/D (Figure 2; numbers 1 and 2 at the top of the aligned EPIYA-C/D block).

### 2.2. Generation of Phosphorylation-Specific Antibodies against EPIYA-Motifs A, B, and C

**Figure 3:**
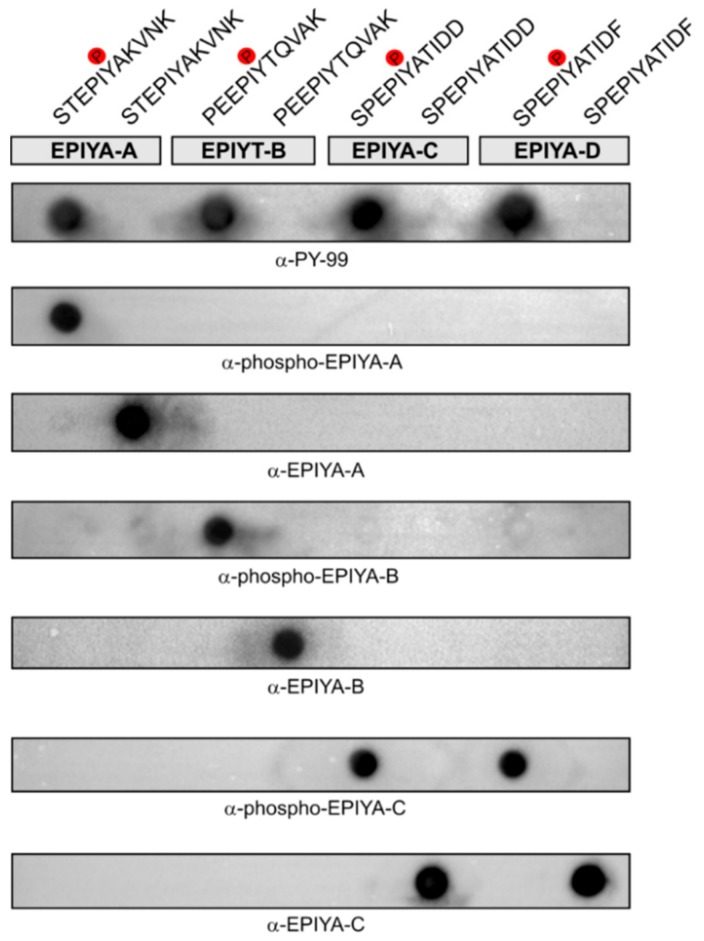
Specific recognition of synthetic 11-mer phospho- and non-phospho-peptides derived from the CagA EPIYA-sites A, B, C, and D by tailor-made antibodies. The indicated phospho- and non-phospho-peptides of the EPIYA-sites were synthesized and blotted onto Immobilon-P membranes using a dotblot system. The peptide sequences cover motifs representing phosphorylation sites, which can be phosphorylated by Src and Abl tyrosine kinases upon infection with *H. pylori*. Membranes with the indicated peptides were probed with the six antibodies generated in this study. Three independent experiments were performed with similar results.

In order to perform a systematic study to investigate the phosphorylation states of CagA, we raised specific antibodies. For this, we synthesized phosphorylated and non-phosphorylated peptides of each EPIYA-site of the model strain 26695. The peptides were designed so that the tyrosine residue that could be phosphorylated was positioned in the middle, flanked by five amino acids on both sides. This resulted in peptides C-STEPIYAKVNK (for EPIYA-A), C-PEEPIYTQVAK (for EPIYA-B), and C-SPEPIYATIDD (for EPIYA-C). A peptide length of 11 amino acids was chosen because peptides of this length are able to generate pan-phosphotyrosine antibodies during immunization, while shorter or longer peptides can result in loss of antibody specificity [48,49,50,51]. The peptides were coupled to a carrier protein, *Limulus polyphemus* haemocyanin (LPH), via an added amino-terminal cysteine residue, and this was used to immunize rabbits, employing two animals per peptide. To select for antibodies specifically recognizing the phosphorylation state of the antigen, all antisera were subjected to immunoglobulin G (IgG) purification columns coupled with the opposing peptide. After this selective removal step, the resulting antibodies were tested for their specificity by dotblots carrying each EPIYA-peptide. As the 11-mer EPIYA-motif C sequence is almost identical to that of EPIYA-motif D (C-SPEPIYATIDF), we also spotted the corresponding EPIYA-D peptides on the dotblots as control. The pan-phosphotyrosine antibody PY-99 was used as a control, as this recognizes all four phosphorylated EPIYA motifs (Figure 3). As shown, the obtained IgG antibodies were not only specific for the EPIYA-A, -B, or -C sequences to which the animals had been immunized, but also for the specific phosphorylation state of these epitopes (Figure 3). The only exception was noted with the antibodies against EPIYA-motif C, which also recognized the closely related EPIYA-D peptides. Thus, the produced antibodies had a strong differentiating specificity for the EPIYA-motifs A, B, and C/D of CagA, and could specifically determine their phosphotyrosine state.

### 2.3. Monitoring of Phosphorylated EPIYA-Motifs in Multiple Strains Upon Infection of AGS Cells

**Figure 4:**
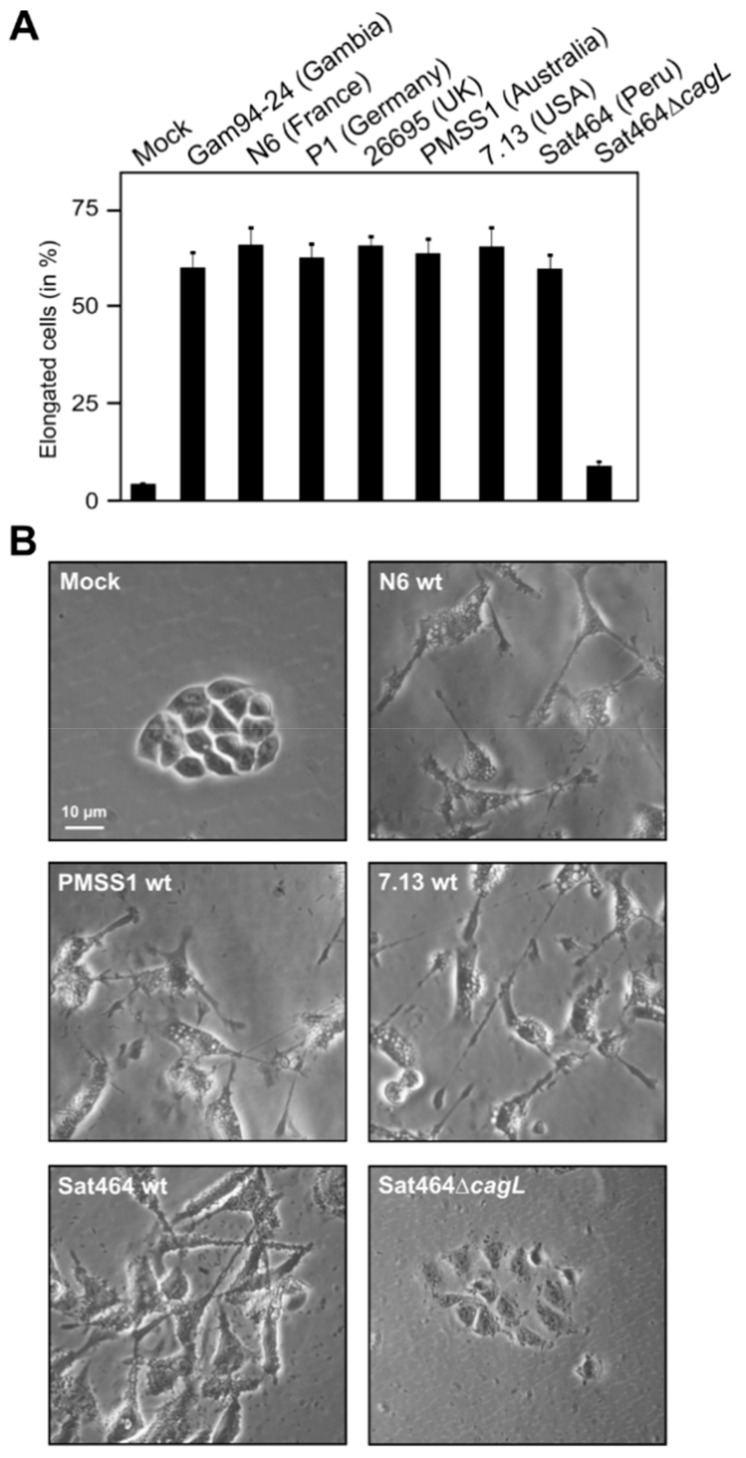
Different clinical *H. pylori* strains induce AGS cell elongation during infection. The indicated ABC-type CagA *H. pylori* strains were used to infect AGS cells for 6 h. (**A**) Quantification of elongated cells was performed in triplicate. Mean values are shown with standard error. (**B**) Phase contrast microscopy of AGS cells infected with some representative strains is shown.

**Figure 5:**
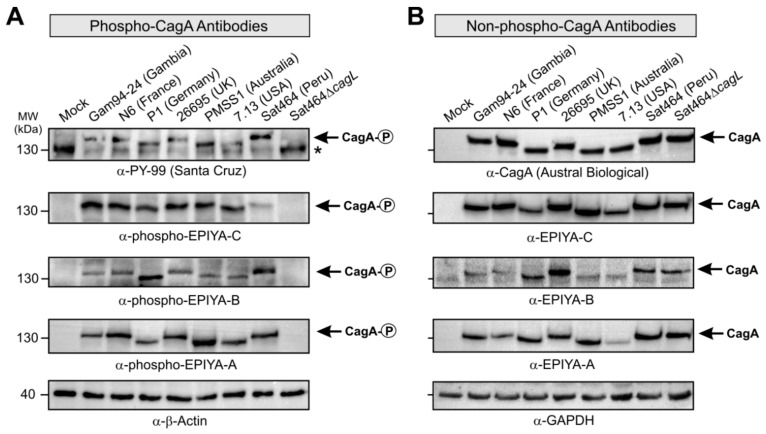
Phosphorylation of CagA EPIYA-motifs during *H. pylori* infection was investigated using specific α-phosphotyrosine antibodies. AGS cells were infected for 6 h with CagA-expressing *H. pylori* strains from countries of five continents as indicated. The samples in Figure 3 were collected after photographing and phenotype quantification. Phosphorylation of CagA was examined using the indicated (**A**) phospho-specific and (**B**) non-phospho-specific EPIYA antibodies with α-PY-99 (Santa Cruz) antibodies as control. Loading of equal amounts of CagA in each sample has been approved by a commercial α-CagA antibody (Austral Biologicals). The phospho-CagA bands of different sizes (arrows) as well as a set of tyrosine-phosphorylated host cell proteins (asterisk) are marked. Three independent experiments were performed with similar results. The corresponding uncropped files are shown in Supplementary Figures S1 and S2, respectively, and the statistics are shown in Supplementary Figure S3.

The raised antibodies were used to confirm that they could be used to study the phosphorylation patterns of CagA^PY^ proteins during in vitro infection with *H. pylori*. For this purpose, seven representative strains carrying the EPIYA A, B, or C-type CagA obtained from patients from different geographic regions were used to infect AGS cells. The elongation phenotype of the infected cells was examined over a time course to indicate successful CagA delivery [52,53,54]. After six h, approximately 55–70% of infected AGS cells exhibited the elongation phenotype, with every individual *H. pylori* strain tested, indicating that large quantities of CagA^PY^ were generated at this time point (Figure 4). The infected cells with attached bacteria were harvested, lysed, and subjected to sodium dodecyl sulfate polyacrylamide gel electrophoresis (SDS-PAGE) and Western blots that were first stained with the pan-phosphotyrosine antibody PY-99 as a positive control (Figure 5A). The different CagA variants revealed slightly different band sizes between 130 and 140 kDa, which is in agreement with the variable amino acid sequences of the encoded proteins (Supplementary Table S1). As expected, the control antibody recognized various bands of tyrosine-phosphorylated host cell proteins at approximately 45, 60, 80, 125, and 170 kDa (see full-size blots in Supplementary Figure S1, asterisks). Exposure of the membranes with a commercial α-CagA antibody confirmed that similar amounts of CagA were loaded in each lane (Figure 5B, top). The other generated non-phospho CagA antibodies also produced reasonable CagA patterns (Figure 5B, see full-size blots in Supplementary Figure S2). The blots were then probed with the three α-phospho EPIYA antibodies, respectively. This resulted in strong bands for phospho-CagA in all seven strains, with very little background signals (Figure 5A). This provides evidence that detectable amounts of individually phosphorylated EPIYA-motifs A, B, and C were present. As a negative control, infection using an isogenic T4SS-deficient Sat464∆*cagL* knockout mutant was included, which was unable to translocate CagA; this control produced no phospho-signals for CagA (Figure 5A, last lanes), further confirming the specificity of our assays. The obtained signals in the blots were quantitated densitometrically as band intensities and these data are shown in Supplementary Figure S3. The data are expressed as relative intensity with respect to the strongest signal in each panel. As expected, this analysis revealed that the relative quantities of bands obtained for the phospho- and non-phospho CagA variants varied among the strains. The quantification based on the two commercial antibodies did not coincide with that of our EPIYA-specific antibodies. For most of the tested strains, one of the EPIYA-motifs was more strongly detected in the phosphorylated form, while another motif was more dominant in its non-phosphorylated form, but which of the motifs was predominately phosphorylated varied per strain. Despite this considerable variation between strains, as an overall trend we detected stronger EPIYA-C signal intensities compared to the other two variants, independent of their phosphorylation state. Moreover, in most strains the signal for the non-phosphorylated EPIYA-C motif in CagA proteins was stronger than its phosphorylated counterpart. This does not necessarily mean that EPIYA-C was more abundant in the phosphorylated compared to the non-phosphorylated form, as it is possible that the EPIYA-C sequence exhibits a higher immunogenicity potential compared to the other EPIYAs, resulting in stronger antibody signals. Since binding efficiency of the antibodies can be also affected by amino acid differences flanking the EPIYA-motif or even by variable positions in other locations of the protein, differences seen between strains or between antibodies have to be interpreted with caution.

### 2.4. Phosphorylation of EPIYA-Motifs in ABC vs. ABD H. Pylori Strains during an Infection Time Course

**Figure 6:**
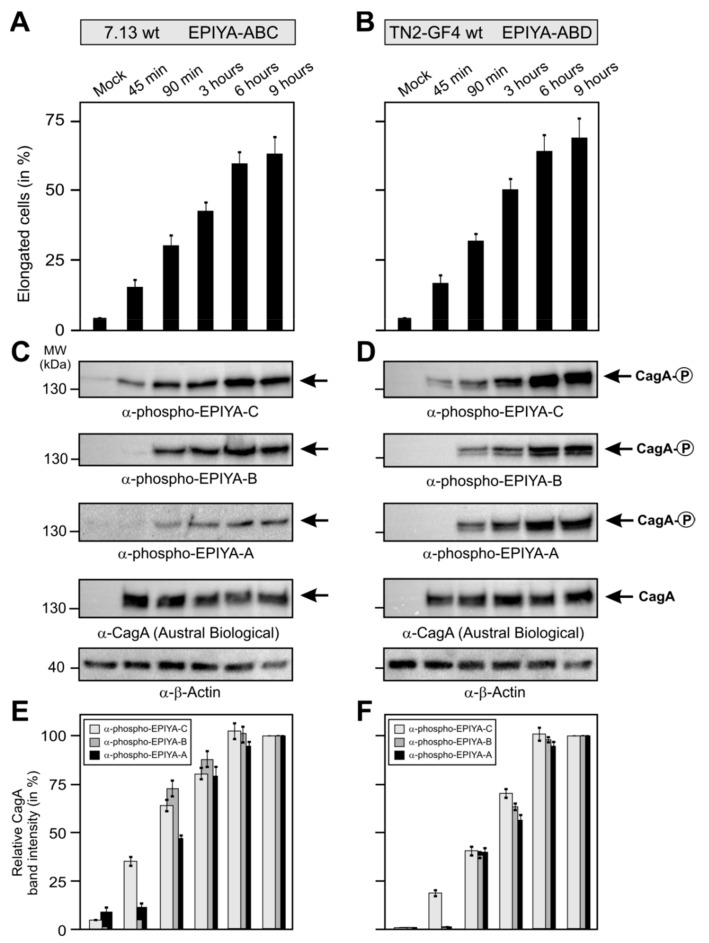
Time-dependent phosphorylation patterns of EPIYA-motifs in *H. pylori* strains during infection of AGS cells. The ABC-type CagA *H. pylori* strain 7.13 (**A**) and ABD-type CagA *H. pylori* strain TN2-GF4 (**B**) were used to infect AGS cells for the indicated times. Quantification of elongated cells was performed in triplicate. Mean values are shown with standard error. (**C**,**D**) Western blotting was performed using the three indicated α-phospho EPIYA antibodies with α-CagA and α-β-Actin as loading controls. (**E**,**F**) Quantification of phospho-CagA band intensities in the above blots was performed in triplicate by densitometry. Mean values are shown with standard error.

Finally, we aimed to study if injected CagA is differentially phosphorylated at certain EPIYA-motifs during a time course of infection. We directly compared *H. pylori* carrying the EPIYA-motif ABC with ABD. For this purpose, we infected AGS cells with the ABC strain 7.13 and ABD strain TN2-GF4-for 45 min up to 9 h (Figure 6). Microscopic monitoring of the infected cells revealed gradually increasing numbers of elongated cells over time, suggesting proper CagA translocation (Figure 6A,B). Accordingly, Western blots stained with the three α-phospho EPIYA antibodies showed increasing intensities for phospho-CagA bands over time for all EPIYAs in both strains (Figure 6C,D). Most notably, the α-phospho EPIYA-C antibody recognized both the phospho-EPIYA-C site in strain 7.13 and the phospho-EPIYA-D site in strain TN2-GF4, which were already detectable as visible bands after 45 min of infection. There were marked differences in band intensities at different time points with the various other antibodies: signals for the phospho-EPIYA-A and -B sites appeared at a later time point than EPIYA-C and became visible only after 90 min, subsequently increasing. Objective quantification of the band intensities of all phospho blots by densitometry confirmed these observations (Figure 6E,F).

## 3. Discussion

The T4SS effector protein CagA and its EPIYA-motifs represent crucial virulence determinants of the gastric pathogen *H. pylori* [2,7,21]. CagA is the first translocated effector protein in the bacterial kingdom for which a host cell kinase was identified [12,13]. The EPIYA-sites in CagA generally share the small amino acid alanine at the +1 position and an isoleucine at the −1 position, similar to the phosphorylation consensus motif EEIYG/E of Src family kinases [12]. In fact, it was demonstrated that Src as well as Abl tyrosine kinases mediate EPIYA phosphorylation both in vivo and in vitro [12,13,14,15,16,55,56,57]. However, progress in this research area is mainly hindered by the lack of standardized commercial EPIYA-specific phospho-CagA antibodies, while little information is available about the specificity of commercial pan-phosphotyrosine antibodies, such as PY-20, PY-99, and PY-100 [43,44]. Consequently, after two decades of research, unambiguous conclusions on CagA phosphorylation patterns in clinical *H. pylori* isolates during infection are still not available. In this study, we demonstrate the generation of three pairs of α-phosphotyrosine antibodies recognizing EPIYA-motifs A, B, and C, respectively, and we have shown that these antibodies display high specificity in recognizing the phosphorylated as well as the non-phosphorylated EPIYA-sites. We confirmed the specificity of each antibody by Western blotting against the corresponding phospho-peptides and excluded cross-reactivity with any non-phospho-peptides. There was only one exception, as antibodies against EPIYA-motif C also recognized the EPIYA-D site. That cross-reactivity could be expected in view of the similarity in amino acid sequence between these sites. Subsequently, infection experiments with *H. pylori* were performed to study the patterns of CagA^PY^ following delivery into host cells. These data demonstrate for the first time that each EPIYA-motif can be phosphorylated during infection with both ABC and ABD strains. Together, these results shed new light on the suitability of such antibodies in *H. pylori* research and pinpoint important new avenues for more detailed analyses on CagA phosphorylation-associated signal transduction events in gastric disease development. For instance, these antibodies may be very useful to study the subcellular localization of individually phosphorylated CagA protein species in cells using high resolution fluorescence microscopy, or they could be applied in immunoprecipitation experiments to identify new host cell binding partners at different phospho-EPIYA motifs.

Remarkably, phosphotyrosine-based signaling is predominant in the domain of higher eukaryotes with many different known kinases involved, such as Src, Abl, EGFR, c-Met, and others, while tyrosine kinases such as the class of BY (bacterial tyrosine) kinases are evolutionary distinct from those in eukaryotes, and some bacteria (including *H. pylori*) do not encode any known tyrosine kinase [58,59,60]. As a consequence, phosphotyrosine-dependent protein–protein interactions have been mainly studied in mammals, and various commercially available pan-phosphotyrosine antibodies were initially designed for mammalian proteins [48,49,50,51]. These antibodies characteristically recognize short synthetic phospho-peptides. For example, previous investigations have used microarrays with 20,000 spotted human phospho-peptides to depict the binding capacities of three widely applied commercial α-pan-phosphotyrosine antibodies, 4G10, PY20, and PY100 [51]. The authors found that each of these antibodies displays a similar binding capacity for phosphotyrosine, but this depends on selected neighboring amino acid residues. Favored binding was observed, with phospho-peptides carrying a leucine at position -1 and proline at position +3 [51]. Interestingly, synthetic phospho-peptides of the CagA EPIYA-sites were shown to be strongly recognized by six commercial pan-phosphotyrosine antibodies [43,44]. Using the dotblot approach, we could demonstrate that two of these antibodies (PY20 and PY99) recognized all three phospho-EPIYA peptides A, B, and C with similar and very strong affinity, confirming that this approach also works for peptides derived from a bacterial effector protein [43,44]. Other commercial pan-phosphotyrosine antibodies mainly recognized phospho-EPIYA peptides A and C (PY100) or predominantly bound to phospho-EPIYA peptide A, with poor binding to C (PY102) or to B and C (PY69). In combination, the overall results revealed that four pan-phosphotyrosine antibodies (PY20, PY69, PY99, and PY100) recognize a broad reservoir of different phospho-EPIYA motifs. However, in studies of *H. pylori* infected host cells, most of these commercial pan-phosphotyrosine antibodies strongly react with host cell proteins of various size ranges as expected, including even proteins of similar size to CagA (~120–140 kDa), which severely limits their use for studying specific CagA phosphorylation events in infected cells.

Despite their limitations, pan-phosphotyrosine antibodies have been widely used in early studies to identify phosphorylated CagA by exploring protein lysates of infected cells separated on one-dimensional SDS-PAGE gels [34,39,40,41,42]. The results obtained in the current study suggest that each of the CagA EPIYA-sites A, B, C, and D can indeed be phosphorylated during infection. However, the conclusions of those earlier observations, based on one-dimensional gels, are widely imperfect because the resulting bands cannot distinguish how many EPIYA-sites are phosphorylated, or which motive is phosphorylated per single CagA molecule. To overcome this, two-dimensional gel electrophoresis was previously employed, which separated different phosphorylated and non-phosphorylated CagA protein spots [23]. Using this strategy and pan-phosphotyrosine antibody PY99, we had already demonstrated that CagA from various strains can be concurrently phosphorylated at one or two EPIYA-motifs per molecule, but never simultaneously at three EPIYA-sites [16,23]. We also determined that Src kinase only phosphorylated EPIYA-C or EPIYA-D, while Abl kinase phosphorylated EPIYA-A, -B, -C, and -D motifs in CagA [16]. Our present study identifies a hierarchic phosphorylation model for CagA starting at EPIYA-C/D, which in our experimental setup was already visible after 45 min of infection, followed by phosphorylation of EPIYA-A or EPIYA-B, which only became visible at later time points. These findings led us to propose that a broad collection of differentially phosphorylated CagA protein molecules are produced in infected host cells, possibly in all possible combinations, which all may have slightly different functions, and this could explain how CagA may coordinate signaling to a high number of different host binding partners [16]. To investigate this assumption in more detail, the EPIYA-specific and phosphorylation-specific CagA antibodies generated in this study will be most useful. However, from the data we present here it cannot yet be concluded whether single CagA molecules can be phosphorylated simultaneously at various positions, which we plan to study in the near future by 2D electrophoresis, as previously described [16]. Moreover, by using Western blotting it cannot be determined how much of the non-phosphorylated CagA detected with infected cells had actually been delivered intracellularly, and how much was residual protein still present inside the adherent bacteria. Lastly, it cannot be excluded that the CagA motifs we detected in host cells with the non-phospho antibodies were originally translocated and present in the phosphorylated form, to be subsequently dephosphorylated by host-derived enzymes. It has been described that host protein tyrosine phosphatases such as SHP1 can display such activity for CagA^PY^ [61]. Thus, it is possible that the findings during *H. pylori* infection of AGS cells shown here represent steady state situations that are the result of changing enzymatic activities in the target cells over time.

The present study focused on the phosphorylation of EPIYA-motifs found in CagA of a diverse set of *H. pylori* strains, for which vast differences were detected, but the findings may be relevant to other bacterial pathogens as well. Tyrosine phosphorylation is a common strategy among bacterial pathogens, although most do not encode their own tyrosine kinases. Enteropathogenic *Escherichia coli*, *Citrobacter rodentium*, *Bartonella henselae*, *Chlamydia trachomatis*, and *Anaplasma phagocytophilum* all exhibit a repertoire of remarkable effector proteins (e.g., Tir, BepD, BepE, BepF, Tarp, and AnkA) carrying EPIYA-like motifs that, similar to CagA, become tyrosine-phosphorylated by host cell kinases upon delivery into host cells [7,9,62,63,64]. Subsequently, these phosphorylated effectors have been shown to bind a selection of cellular interaction partners with Src homology 2 (SH2) domains to manipulate host cell signaling (for reviews see previous work [9,62,63]). Future investigations will likely focus on the phosphorylation of EPIYA-motifs in each of these bacterial effector proteins, to elucidate any resulting signal transduction events in more detail [2,4,17,38,64,65]. For such studies, the generation of antibodies highly specific for each EPIYA-motif and their phosphorylation state is necessary, which we have demonstrated here to be feasible. In the future, this would help to better understand the role of single EPIYA-motifs for bacterial effector protein function and possibly allow correlations and risk predictions for the occurrence of different microbial diseases.

## 4. Materials and Methods

### 4.1. Bioinformatic Analysis of CagA Amino Acid Sequences

The CagA amino acid sequence from *H. pylori* strain 26695 (accession number WP_000180747) was retrieved from GenBank and used as a query in BlastP against the *H. pylori* taxon (Taxid: 210) at NCBI. Only CagA entries bearing the EPIYA-motif ABC or ABD type were selected for analysis (Supplementary Table S1). The amino acid sequences were aligned in Muscle [66] and manually edited in Jalview [67]. The evolutionary history of the CagA amino acid sequences was inferred using the Neighbor-Joining method [68] with a statistical robustness for the associated taxa of 1000 replicates using the bootstrap test [69]. The evolutionary distances were computed using the JTT matrix-based method, and the rate variation among sites was obtained with a gamma distribution (shape parameter = 2) [70]. The evolutionary analyses were conducted in MEGA7 [71] and the final tree was visualized in Itol [72].

### 4.2. H. pylori Strains and Culturing Conditions

The wild-type *H. pylori* strains Gam94-24, N6, P1, 26695, PMSS1, 7.13, TN2-GF4, and Sat464 were typical type-I isolates expressing CagA (Supplementary Table S1). The bacteria were grown on horse serum gonococcal (GC) agar plates containing vancomycin (10 μg/mL), nystatin (1 μg/mL), and trimethoprim (5 μg/mL), and in the case of mutants kanamycin (8 μg/mL), as described previously [73,74]. Mutagenesis of the *cagL* gene was performed by insertion of a kanamycin resistance gene cassette as described [75]. All antibiotics were obtained from Sigma-Aldrich. Bacteria were grown at 37 °C for 2 days in an anaerobic jar containing a Campygen gas mix of 5% O_2_, 10% CO_2_, and 85% N_2_ (Oxoid, Wesel, Germany) [76,77].

### 4.3. Production of Phospho-and Non-Phospho-Specific EPIYA Antibodies

The following peptide sequences were synthesized by Jerini (Berlin, Germany): C-STEPIYAKVNK (EPIYA-A), C-STEPI(pY)AKVNK (phospho-EPIYA-A), C-PEEPIYTQVAK (EPIYA-B), C-PEEPI(pY)TQVAK (phospho-EPIYA-B), C-SPEPIYATIDD (EPIYA-C), and C-SPEPI(pY)ATIDD (phospho-EPIYA-C). The peptides C-SPEPIYATIDF (EPIYA-D) and C-SPEPI(pY)ATIDF (phospho-EPIYA-D) were synthesized for use of as controls. Each of these EPIYA peptides was purified by High Performance Liquid Chromatography (HPLC) and their purity was approved by mass spectrometry analysis (Jerini, Berlin, Germany). All peptides were synthesized with an additional amino-terminal cysteine residue to which *Limulus polyphemus* haemocyanin (LPH) carrier protein was conjugated (Biogenes, Berlin, Germany). Two rabbits were immunized with each conjugated peptide according to a standard protocol by Biogenes using an immunization schedule summarized in Table 1. The resulting antisera were used to select phospho-specific antibodies by affinity-removal of the corresponding non-phospho-peptide of each EPIYA-motif bound to a column. Conversely, the obtained non-phospho antibodies were affinity-purified using columns against the corresponding phospho-peptide. These antibodies were prepared on a customer basis and purified by Biogenes.

### 4.4. Dotblot Analysis

Dotblots were prepared by spotting 20 μg of each peptide in 1 mL of transfer buffer (192 mM Glycine; 20 mM Tris-HCl, pH 8.4; 0.1% SDS; 20% Methanol) onto Immobilon-P membrane (Millipore, Darmstadt, Germany) using the BioDot apparatus (BioRad, Munich, Germany) [78,79]. The resulting dotblots were dried at 37 °C and subjected to antibody detection by Western blots, as described below.

### 4.5. Host Cell Culture, Infection Assays, and Elongation Phenotype Quantitation

The human gastric adenocarcinoma cell line AGS (ATCC CRL-1739™) was cultured on 6-well plates in RPMI1640 medium (Invitrogen, Karlsruhe, Germany) supplemented with 10% fetal calf serum (Invitrogen) and a penicillin/streptomycin cocktail (Sigma-Aldrich, Taufkirchen, Germany). The cells were grown in 5% (*v*/*v*) CO_2_ at 37 °C [80,81]. When the cells reached about 75% confluency, they were washed two times with Phosphate Buffered Saline (PBS) and fresh RPMI1640 medium without antibiotics was added [82,83]. Cells were serum-deprived overnight and infected with *H. pylori* at a multiplicity of infection (MOI) of 50 for 6 h. After infection, the cells were harvested in ice-cold PBS containing 1 mmol/l Na_3_VO_4_ (Sigma-Aldrich). Using an Olympus IX50 phase contrast microscope, the elongated AGS cells were quantitated in 10 different 0.25-mm^2^ fields per experiment [84,85]. All experiments were done in triplicate.

### 4.6. SDS-PAGE and Western Blotting

Infected AGS cells were washed twice with PBS to remove unbound bacteria, and then the cells were harvested with attached bacteria, pelleted, and resuspended in equal amounts of 2× SDS-PAGE buffer. Proteins were boiled for 5 min and then subjected to SDS-PAGE. The proteins were resolved in 6% polyacrylamide gels and blotted onto Immobilon-P membranes (Millipore). Membranes were blocked in Tris-buffered saline with Tween20 (TBST) buffer with 3% bovine serum albumin (BSA) or 5% skim milk for 1 h at room temperature followed by incubation with the generated α-phosphotyrosine and non-phospho CagA antibodies [86,87]. As controls, we used a rabbit polyclonal α-CagA antibody (Austral Biologicals, San Ramon, CA, USA) and the mouse monoclonal pan-phosphotyrosine antibody PY-99 (Santa Cruz, Dallas, TX, USA) according to the instructions by the manufacturers. Phosphorylated and non-phosphorylated CagA proteins were detected using horseradish peroxidase-conjugated anti-rabbit or anti-mouse polyvalent goat immunoglobulin secondary antibodies visualized by the Amersham enhanced chemiluminescence (ECL) Prime Western blot kit (GE Healthcare, Munich, Germany), as described previously [88,89].

### 4.7. Statistical Analysis

The intensities of the Western blot signals, derived from three independent experiments, were measured and quantitated densitometrically by the Image Lab™ software (Bio-Rad). For each α-phosphotyrosine and α-non-phospho CagA/EPIYA Western blot, shown in Figure 5 and Figure 6C,D, the strongest band on the gels was set to 100% and the relative intensities of every single band on the respective blot were calculated (Supplementary Figure S3 and Figure 6E,F). The error bars for quantifying relative band intensities in Supplementary Figure S3 and number of elongated cells shown in Figure 4 represent the standard error of the mean (SEM) calculated with Excel software.

## 5. Conclusions

All commercially available pan-phosphotyrosine antibodies were originally produced to detect a wide array of phosphotyrosines in multiple mammalian proteins, but were not optimized for usage in bacterial effector proteins. Here we generated 11-mer phospho- and non-phospho-peptides from the EPIYA-motifs A, B, and C of *H. pylori* CagA, and produced three phospho-specific and three non-phospho-specific polyclonal antibodies in rabbits. We demonstrated that these antibodies specifically detect the corresponding phosphorylated and non-phosphorylated EPIYA-motifs. Interestingly, the EPIYA-C antibodies also recognized the closely related East-Asian motif EPIYA-D in vitro. Furthermore, we showed that each EPIYA-motif can be efficiently phosphorylated during infection with *H. pylori* ABC and ABD strains in vivo. Together, we present here the first complete set of phospho-specific antibodies for a bacterial effector protein, which are new beneficial tools to collect important data for cataloguing CagA phosphorylation events, disease development in the human stomach and gastric cancer signal transduction.

## Figures and Tables

**Table 1:** Time scheme of rabbit immunization.

Day	Step
0	Peptide synthesis and LPH coupling
0	Collection of pre-immune serum
0	First immunization of 2 rabbits
7	First boost
14	Second boost
28	Third boost
42	Bleeding, fourth boost
56	Fifth boost
70	Sixth boost
77	Bleeding
105	Final bleeding, serum IgG purification

## Supplementary Materials

The following are available online at https://www.mdpi.com/2072-6694/11/8/1163/s1. Table S1: Worldwide CagA protein sequences analyzed in the study, Figure S1: CagA EPIYA-motif phosphorylation during *H. pylori* infection of AGS cells was investigated using the indicated α-phosphotyrosine antibodies, Figure S2: CagA expression during *H. pylori* infection of AGS cells was investigated using the indicated α-CagA and α-EPIYA antibodies, Figure S3: Statistics of CagA EPIYA-motif phosphorylation and expression of CagA during *H. pylori* infection of AGS cells.
