# Supplementary material for: Tailor-Made Detection of Individual Phosphorylated and Non-Phosphorylated EPIYA-Motifs of Helicobacter pylori Oncoprotein CagA
Source: Cancers (Basel). 2019 Aug 13;11(8):1163. doi: 10.3390/cancers11081163 (PMC6721621; doi:10.3390/cancers11081163)
Supplement: Supplementary file 1 [file cancers-11-01163-s001.zip › Supplementary Figures S1-S3.pdf]

Supplementary Materials

Tailor-Made Detection of Individual Phosphorylated and Non-Phosphorylated EPIYA-Motifs of *Helicobacter pylori* Oncoprotein CagA

Suneesh Kumar Pachathundikandi, Andrés Julián Gutiérrez-Escobar and Nicole Tegtmeyer

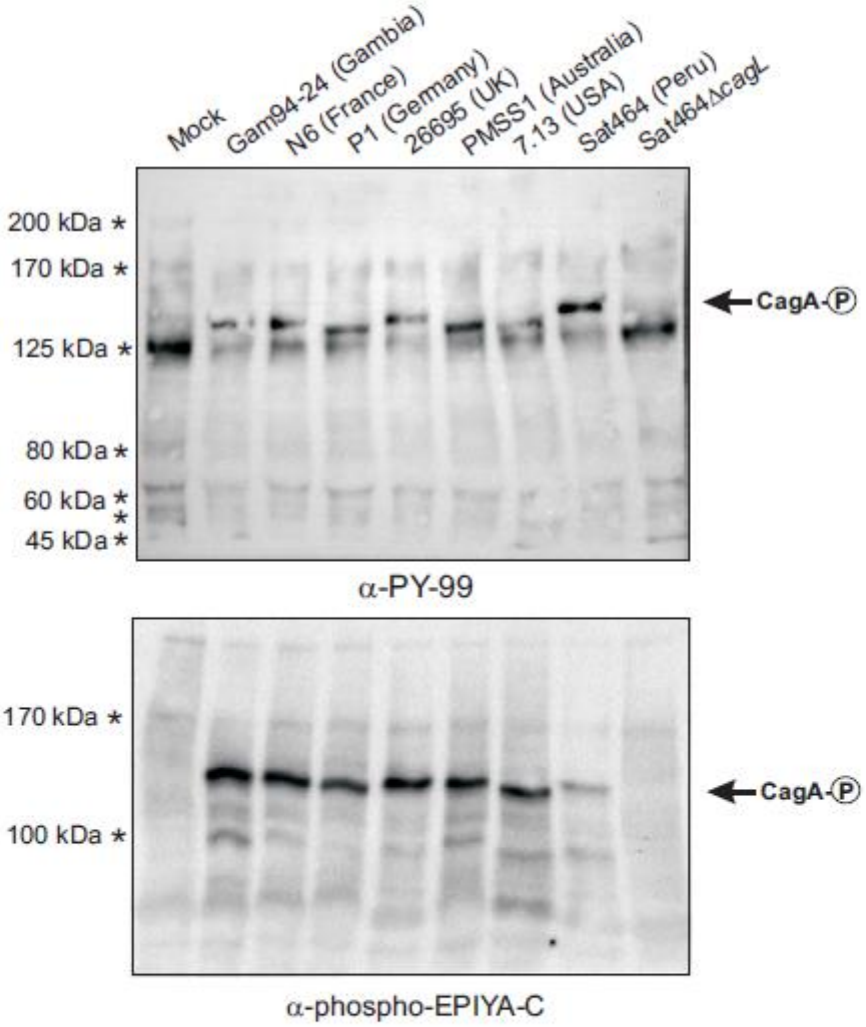

Figure S1. *Cont.*

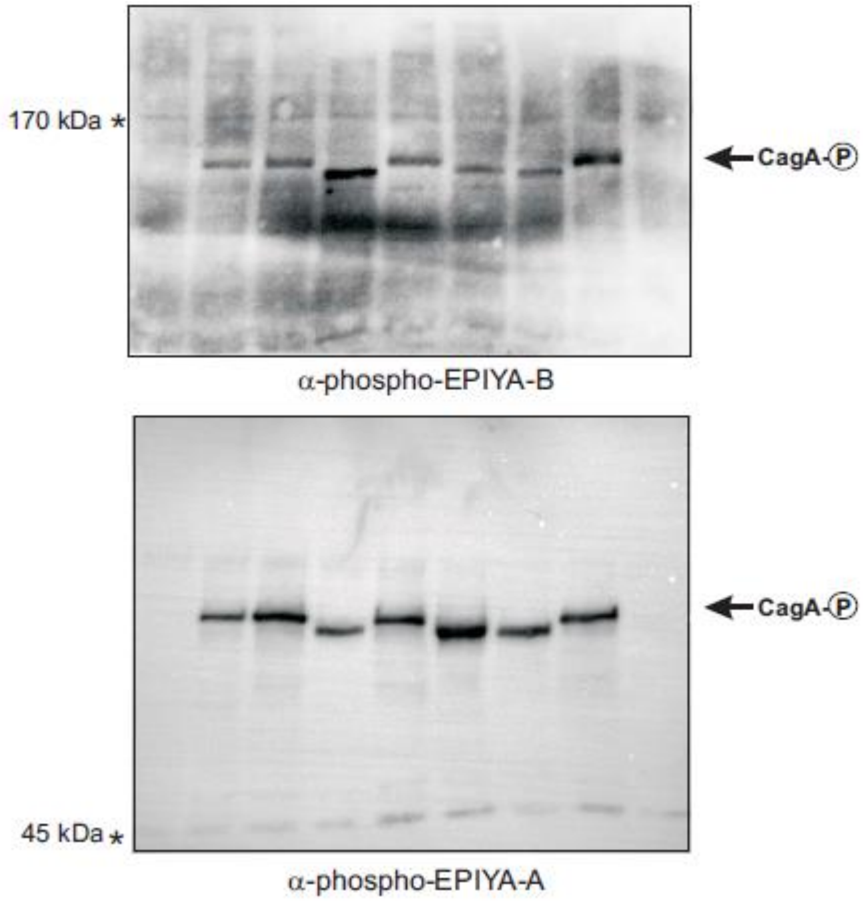

Figure S1. CagA EPIYA-motif phosphorylation during *H. pylori* infection of AGS cells was investigated using the indicated α-phosphotyrosine antibodies. Full-size blots of Figure 5A are shown. The phospho-CagA bands are labelled with arrows. A set of tyrosine-phosphorylated host cell proteins also appeared, which are marked with asterisks and sizes given in kDa.

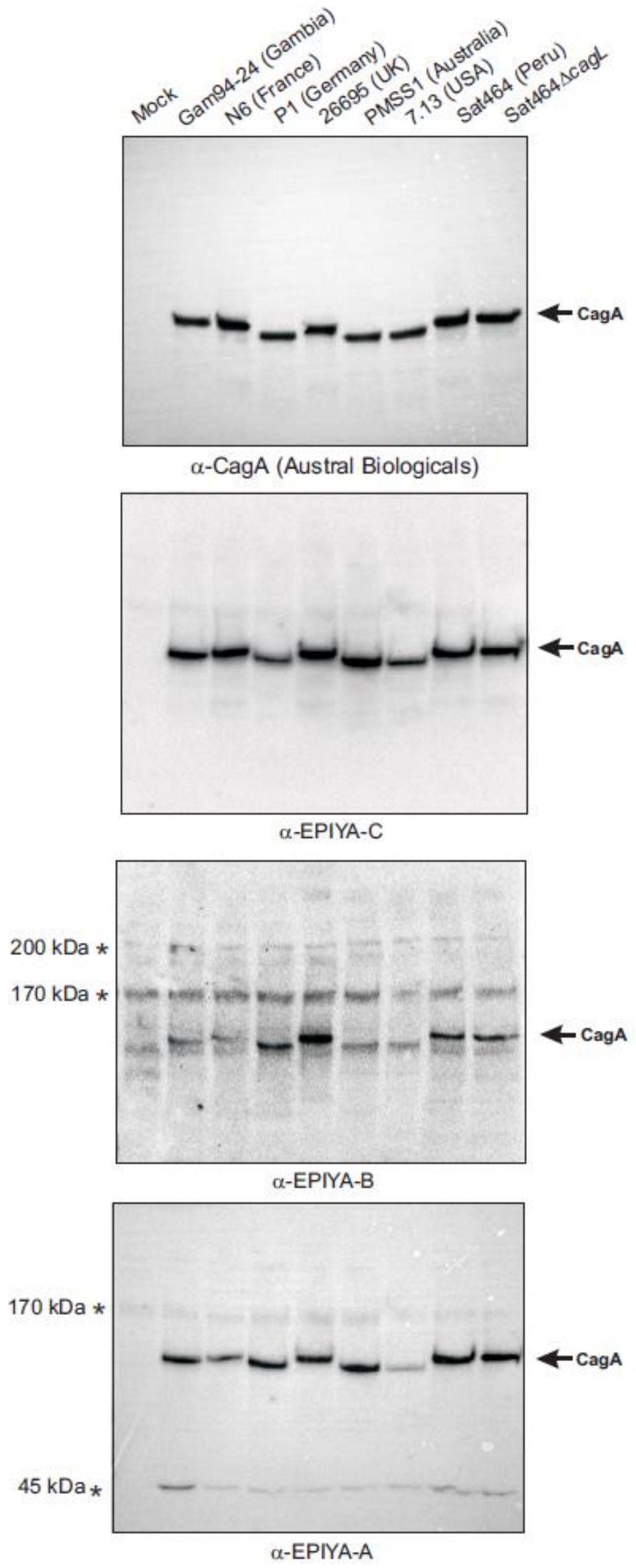

Figure S2. CagA expression during *H. pylori* infection of AGS cells was investigated using the indicated α-CagA and α-EPIYA antibodies. Full-size blots of Figure 5B are shown. The non-phospho-CagA bands are labelled with arrows. A set of cross-reacting host cell proteins are also seen, which are marked with asterisks and sizes in kDa.

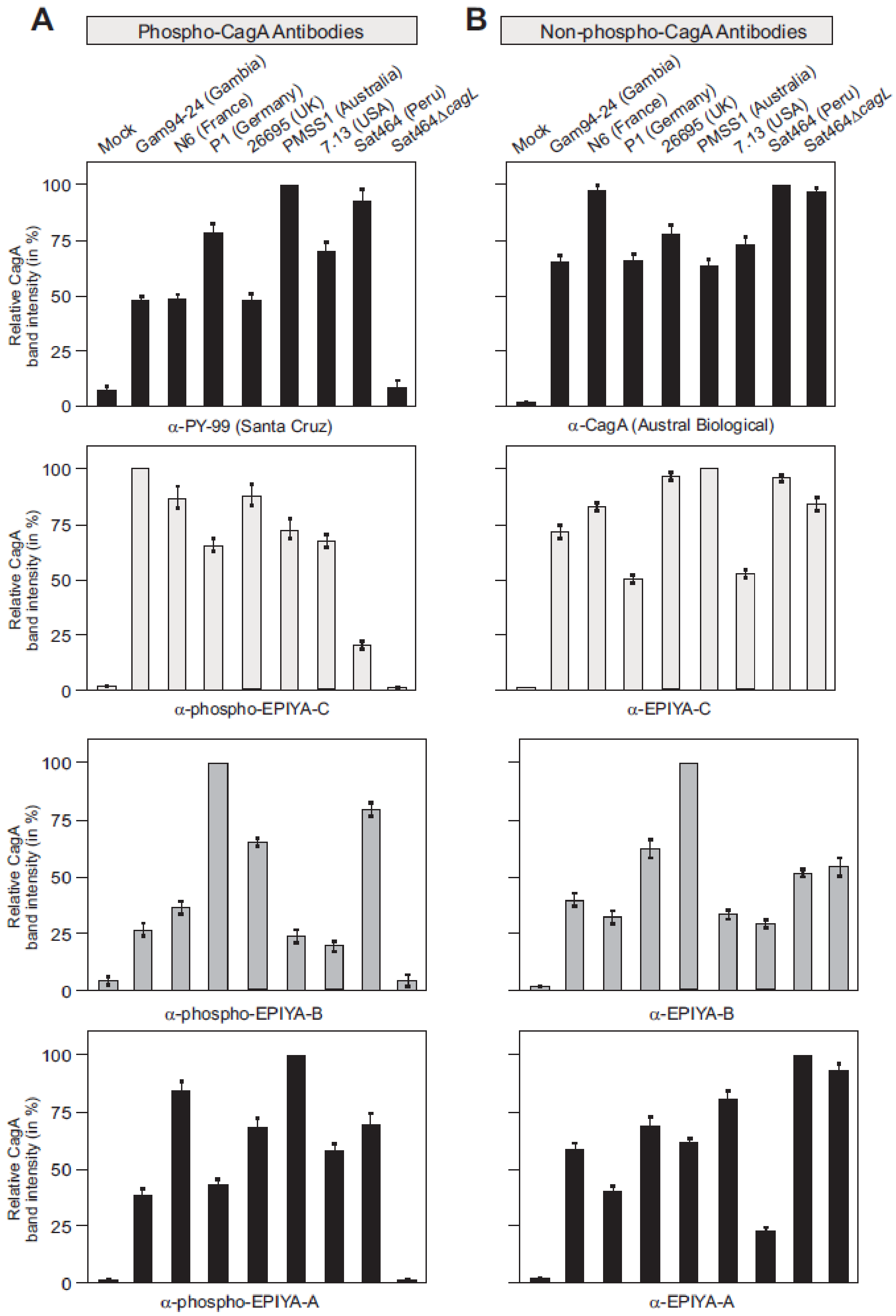

Figure S3. Statistics of CagA EPIYA-motif phosphorylation and expression of CagA during *H. pylori* infection of AGS cells. Band intensities of samples using the indicated specific α-phosphotyrosine (**A**) and α-CagA/EPIYA (**B**) antibodies shown in Figure 5 were measured by densitometry. The strongest band on each blot was set 100%. Three independent experiments were performed with similar results.

© 2019 by the authors. Licensee MDPI, Basel, Switzerland. This article is an open access article distributed under the terms and conditions of the Creative Commons Attribution (CC BY) license (http://creativecommons.org/licenses/by/4.0/).
